# The Interplay Between Sleep Apnea and Postpartum Depression

**DOI:** 10.3390/neurolint17020020

**Published:** 2025-01-28

**Authors:** Antonino Maniaci, Luigi La Via, Mario Lentini, Basilio Pecorino, Benito Chiofalo, Giuseppe Scibilia, Salvatore Lavalle, Antonina Luca, Paolo Scollo

**Affiliations:** 1 Department of Medicine and Surgery, University of Enna “Kore”, 94100 Enna, Italy; basilio.pecorino@unikore.it (B.P.); benito.chiofalo@unikore.it (B.C.); salvatore.lavalle@unikore.it (S.L.); antonina.luca@unikore.it (A.L.); paolo.scollo@unikore.it (P.S.); 2 Department of Anesthesia and Intensive Care, University Hospital Policlinico “G.Rodolico—San Marco”, 95123 Catania, Italy; luigilavia7@gmail.com; 3 Giovanni Paolo II Hospital, ASP 7, 97100 Ragusa, Italy; marlentini@tiscali.it (M.L.); giuseppe.scibilia@unikore.it (G.S.)

**Keywords:** obstructive sleep apnea, pregnancy disorders, postpartum depression, maternal health, sleep disorders, sleep fragmentation, intermittent hypoxia

## Abstract

The complicated association between sleep apnea and postpartum depression (PPD), two diseases that can have a major influence on a mother’s health and well-being, is examined in this thorough review. An increasing number of people are realizing that sleep apnea, which is defined by repeated bouts of upper airway obstruction during sleep, may be a risk factor for PPD. The literature currently available on the frequency, common risk factors, and possible processes relating these two disorders is summarized in this study. We investigate the potential roles that sleep apnea-related hormone fluctuations, intermittent hypoxia, and fragmented sleep may play in the onset or aggravation of PPD. We also talk about the difficulties in identifying sleep apnea in the postpartum phase and how it can affect childcare and mother–infant attachment. The evaluation assesses the effectiveness of existing screening techniques, available treatments, and how well they manage both illnesses at the same time. Lastly, we identify research gaps and suggest future lines of inquiry to enhance maternal health outcomes.

## 1. Introduction

Sleep apnea and postpartum depression are two major maternal health conditions that fundamentally impact outcomes for both mothers and children. While these conditions have traditionally been studied as separate entities, emerging evidence suggests that important interactions between these disorders call for comprehensive investigation. Sleep apnea is a condition characterized by repeated upper airway obstruction during sleep [1]. It has been reported in 2–10% of the general adult population, and its prevalence increases during pregnancy and the postpartum period due to altered physiological conditions and hormonal changes [2,3]. Postpartum depression affects 10–15% of mothers within the first year following delivery and is manifested by maintained depressive symptoms, anxiety, and difficulty in bonding with the infant [4,5]. The coexistence of such conditions introduces significant clinical challenges. Women with both disorders are at an increased risk for pregnancy complications, including preeclampsia and gestational diabetes [6,7]. Beyond acute concerns regarding maternal health, such conditions can substantially affect the development of a child, delaying their cognitive and emotional development, impairing the attachment between mother and infant, and increasing the risk of behavioral problems in childhood [8]. The interplay of these two conditions may, thus, render a very hostile environment for maternal recovery and the development of the infant. Recent studies have highlighted several mechanisms that could explain the link between the disorders, as follows: disrupted sleep is common to both conditions and impacts mood regulation and maternal caregiving capacity [9]. Physiological changes, such as hormonal fluctuations, weight gain, and fluid retention, contribute to alterations in the upper airways and disturbances in mood [10,11]. Additional evidence for a shared pathophysiological basis comes from the recognition of shared risk factors, including obesity, inflammatory processes, and neuroendocrine dysregulation [12]. These factors interact in a complex manner with each other and can lead to a vicious circle of cause and effect, which may lead to the maintenance of both conditions [13,14]. Healthcare providers also have significant challenges in managing these conditions. Symptom overlap with normal pregnancy changes leads to diagnostic difficulties and, thus, frequent underdiagnoses of sleep apnea in women. Disrupted maternal sleep is often attributed to normal postpartum adjustment and may obscure more serious underlying sleep disorders [15,16,17].

Postpartum blues (postnatal baby blues) describe an affective disorder that affects 80% of mothers within 2 weeks of childbirth, characterized by spontaneous resolution, but can develop in 10–15% of mothers after 2 weeks postpartum, with the persistence of symptoms beyond 2 weeks in duration and more severe symptoms [16]. Healthcare providers need to differentiate these disorders based on their unique timelines and symptom severities. Postpartum blues is characterized by mild mood changes, tearfulness, and anxiety, which peak around day 5 of the postpartum period and resolve by day 14. In comparison, the definition of PPD encompasses a decreased mood, anhedonia, important anxiety, and impairment lasting at least two weeks, usually occurring during the first three months postpartum [18]. It is important to recognize these different temporal patterns and symptom profiles for accurate diagnosis and intervention timing.

These diagnostic barriers become further compounded by treatment barriers, including stigma against mental health, poor access to specialized services, and a lack of resources. Current clinical trends support early intervention with comprehensive models of care. Indeed, there has been a growing move toward integrating screening protocols and combined treatment strategies to address comorbidity within healthcare systems [19]. Implementation in practice has often proved challenging in many settings due to resource constraints and the difficulty in coordinating care through multiple specialties. This calls for the realization of an effective intervention by understanding the complex relationship between sleep disorders and maternal mental health within the real constraints of delivering care to new mothers. We review the bidirectional relationship between sleep apnea and PPD, considering existing diagnostic and treatment approaches to define the main areas for future research and clinical improvement. It is important to outline such interactions for the elaboration of successful interventions that may favorably affect both conditions and, subsequently, improve the outcomes in mothers and their children. By searching for the latest studies and clinical evidence, we seek to provide healthcare providers with practical insights into how to identify and manage these concurrent conditions in the postpartum population. We review the epidemiology, common pathophysiological mechanisms, current diagnostic challenges, and various treatment approaches of both conditions. The current issues regarding maternal and infant health outcomes are evaluated considering both the short-term and long-term consequences of these conditions on maternal and infant health outcomes when they co-occur. Finally, we discuss the emerging directions for research and possible new therapeutic strategies that may lead to improved care for this vulnerable population.

With this systematic review, we hope to further the knowledge of the interplay between sleep apnea and PPD during the postpartum period, which, in turn, could lead to better clinical practice for the improvement of health outcomes in mothers and babies. This is of the utmost importance for more effective integrated approaches to maternal health during the critical postpartum period.

## 2. Materials and Methods

### 2.1. Literature Search Strategy

We conducted a systematic review of the literature via the online databases PubMed, EMBASE, and Cochrane for articles published up to July 2024, without time restriction. The search strategy included a combination of Medical Subject Headings (MeSHs) and free-text terms to highlight the concepts of “obstructive sleep apnea” and “postpartum depression.” The search strategy combined MeSH terms and keywords using the following Boolean operators: (“obstructive sleep apnea” OR “sleep disordered breathing” OR “sleep disorders”) AND (“depression” OR “post-partum” OR “postpartum depression”) AND (“pregnancy” OR “gestational” OR “maternal” OR “perinatal” OR “delivery”) AND (“CPAP therapy” OR “treatment” OR “management”).

We included articles reporting data on the epidemiology, pathophysiology, or maternal and fetal outcomes in patients with PPD or sleep disorders and OSA.

We applied filters to include only articles written in English and studies involving human subjects. Further, we selected studies based on the following intended outcomes:

- Maternal outcomes: Sleep apnea symptoms, depressive symptoms, pregnancy complications (preeclampsia and gestational diabetes), adherence to interventions, and maternal mental health.
- Fetal outcomes: Birth weight, gestational age, Apgar scores, neonatal health, and cognitive or developmental milestones.

Studies that did not report these outcomes or focused solely on unrelated comorbidities were excluded.

We included observational studies, randomized controlled trials (RCTs), case–control studies, cohort studies, and cross-sectional studies. Reviews, meta-analyses, and clinical guidelines were also considered to provide context and discuss current recommendations. Case reports, editorials, and commentaries were excluded from the review.

The following PICOTS framework was adopted for the literature search and data extraction:

P (Population): Pregnant and postpartum women;
I (Intervention): Sleep assessments, CPAP therapy, and psychosocial interventions (peer support);
C (Comparison): Standard care or no intervention;
O (Outcomes): Primary—sleep apnea symptoms and depressive symptoms; secondary—maternal data such as pregnancy complications (preeclampsia and gestational diabetes) and treatment adherence (medical treatment, CPAP use, and psychotherapy) and fetal data such as neonatal health findings (birth weight and gestational age), Apgar scores, and developmental outcomes (e.g., cognitive and emotional milestones)
T (Timing): Pregnancy through to first year postpartum;
S (Setting): Hospital-based antenatal clinics, medical centers, and population-based cohorts.

### 2.2. Review Process and Conflict Resolution

Two reviewers independently screened titles and abstracts for relevance, followed by a full-text review to determine their eligibility. Discrepancies were resolved through agreement or by involving a third senior reviewer. Data extracted from the selected studies included author(s), year of publication, study design, sample size, participant characteristics, diagnostic criteria for OSA, outcomes measured, and key findings.

## 3. Results

**Figure 1:**
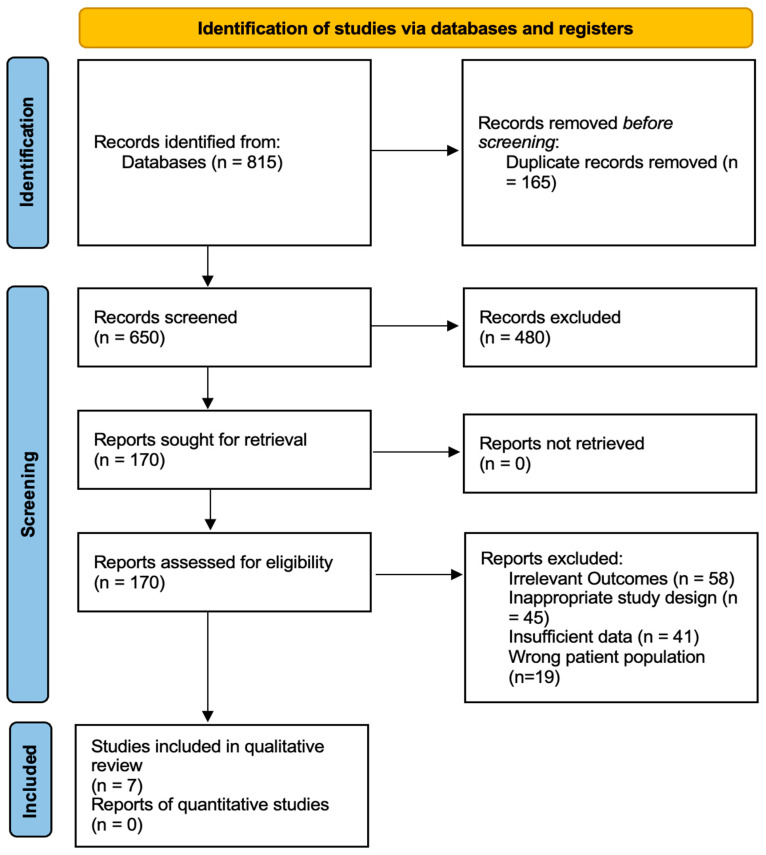
PRISMA flow diagram for literature research protocol.

The initial screening identified 815 records. After removing 165 duplicates, 650 articles were screened by title and abstract. Of these, 480 were excluded based on the predefined criteria, leaving 170 articles for full-text assessment. Full-text screening eliminated 163 articles for the following reasons: irrelevant outcomes (*n* = 58), inappropriate study design (*n* = 45), insufficient data (*n* = 41), and wrong patient population (*n* = 19). This resulted in a final seven studies that met all the inclusion criteria (Figure 1).

We found three prospective studies, two cross-sectional studies, one randomized controlled trial, and one longitudinal cohort study. The samples varied from 105 to 1,577,632 participants. The studies mainly included pregnant and postpartum women, with the screening tools included the Edinburgh Postnatal Depression Scale (EPDS), Berlin Questionnaire, polysomnography, and various sleep assessments. Snoring during pregnancy occurred in 35% of women [19] and was associated with an increased risk of gestational hypertension (OR 2.3, CI 95% 1.4–4.0) and preeclampsia (OR’s > 2) (CI values not reported). Research with a longitudinal perspective on pregnancy demonstrated modifications in outcomes based on timing; OSA frequency increased from early to late pregnancy, and the trajectories of depressive symptoms postpartum were distinct over breastfeeding duration. Peer support was associated with a 40% reduction in risk of PPD in all intervention studies. Limitations that were commonly observed among these studies included self-reporting, selection bias, and a limited generalizability. Considering levels of evidence ranging from Level I to III, most studies provided Level II evidence. The main findings are represented in Table 1.

## 4. Epidemiology, Prevalence, and Shared Risk Factors

### 4.1. Prevalence of Sleep Apnea in Postpartum Women

The exact prevalence of sleep apnea is difficult to define, because screening techniques, diagnostic standards, and evaluation timing can vary. According to Dominguez et al.’s systematic review and meta-analysis, the prevalence of OSA in pregnancy varied from 3.7% to 37.3% [25]. This large variation is explained by variations in the diagnostic standards and the gestational age at which the women were examined. Crucially, the prevalence of OSA tends to rise with the progression of pregnancy, peaking in the third trimester. Based on polysomnography, a study by Bourjeily et al. found that 3.7% of women developed OSA 6–8 weeks after giving birth [26]. On the other hand, the prevalence of symptoms related to disordered sleep breathing was substantially greater, ranging from 20% to 30% when employing questionnaire-based screening tools [20].

### 4.2. PPD Prevalence and Risk Factors

Postpartum women are more likely to have sleep apnea due to a combination of factors such as fluid retention, hormone changes that alter upper airway patency, and residual weight gain after pregnancy [11]. In addition, the symptoms related to OSA may worsen because of sleep fragmentation brought on by baby care obligations. Among women globally, PPD is a serious public health issue. According to a thorough meta-analysis conducted by O’Hara and McCabe, 13% of women experience PPD in the first year following childbirth [4]. This estimate agrees with the range of 10% to 15% that is frequently mentioned in public health bulletins and professional guidelines [27]. In addition, PPD incidence can vary among various groups and cultural settings. Hahn-Holbrook et al. reported that greater PPD rates are seen in low- and middle-income countries [24]. Another important consideration is the time of the beginning of PPD. While PPD may occur later in the first year following childbirth, some women notice the onset of depression symptoms during the first few weeks postpartum. The prevalence of heightened depression symptoms was 14% at two months postpartum, 15% at six months, and 13% at twelve months according to longitudinal research by Putnick et al. [21]. Numerous studies have investigated the relationship between disordered sleep breathing and PPD, while precise co-occurrence rates are yet unknown. According to a study by Okun et al., PPD was found to be more common in women with a history of depression who had inadequate sleep throughout pregnancy [9]. Redhead et al. looked at the connection between early PPD symptoms and symptoms of sleep disorders. It was discovered that, after two weeks postpartum, women who reported symptoms of OSA were 2.8 times more likely to experience symptoms of depression [22]. Bourjeily et al. conducted a prospective study to look at the relationship between depression and OSA during pregnancy and the postpartum phase [26], finding a higher prevalence of depression at 6 months postpartum (33%), compared to those without OSA (19%). These prevalence patterns are important to understand in relation to healthcare resource allocation and clinical practice. These relatively high rates of co-occurrence would suggest that the screening of one condition should prompt an evaluation for the other, thus assuring early intervention and better outcomes for the affected mothers and their infants [27,28].

## 5. Pathophysiological Mechanisms of Sleep Apnea and PPD

### 5.1. Physiological Changes During Pregnancy

**Figure 2:**
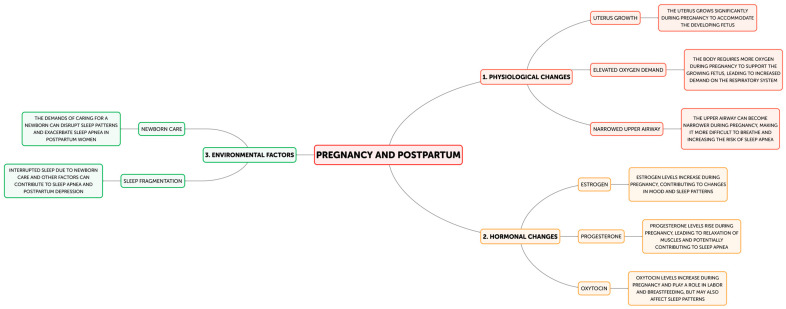
Pathophysiology of PPD and sleep apnea.

Despite multiple physiological changes occurring during pregnancy and postpartum (Figure 2) affecting respiratory function and mood, the expanded uterus raises the diaphragm up to 4 cm and decreases the fetid residual capacity by 10–25% (from about 1.7 L to about 1.35 L) [29]. Along with a 20–30% increase in oxygen consumption and hormonal changes, this leads to OSA susceptibility [30]. Increased estrogen levels (6000–30,000 pg/mL late pregnancy) cause upper airway mucosal edema due to increased mucosal gland secretion and vascular permeability. In addition, the increased level of progesterone during this time, specifically 100–200 ng/mL during late pregnancy, modulates the sensitivity of the respiratory centers, such that an abrupt decline in them postpartum could lead to shortness of breath [30,31]. These hormonal fluctuations also influence neurotransmitter systems. In particular, estrogen influences serotonergic pathways and GABA receptor expression, while progesterone metabolites influence GABA receptor functionality, impacting both respiratory and mood regulation [32]. Further, postpartum fluid shifts may result in the exacerbation of upper airway edema [31] and mood and sleep disruptions due to infant care [32]. Both disorders are characterized by changes in autonomic nervous system activity. Though an increased sympathetic and reduced parasympathetic tone operate in these conditions [33,34], such autonomic changes do promote bronchodilation through relaxation of the bronchial smooth muscle, which could provide a compensatory response in favor of breathing. However, this persistent autonomic imbalance may have other physiological effects that would need attention, such as cardiovascular effects [33,34,35].

In addition, pregnancy complications, including preeclampsia and gestational diabetes, predispose to an increased vulnerability for both disorders [36,37,38,39,40], potentially related to the common pathways of inflammation and vascular abnormalities.

### 5.2. Hormonal and Hypothalamic–Pituitary–Adrenal (HPA) Axis Pathways

The hormonal milieu makes all the difference in the development of these conditions [23,41,42]. Progesterone, the postpartum levels of which fall dramatically, acts to modulate sleep and respiratory control [43], whereas the effects of estrogen on the upper airway mucosa increase the risk of obstruction of the upper airways [44]. In particular, the progesterone level falls dramatically during the postpartum period from 100–200 ng/mL during pregnancy to less than 2 ng/mL within 24 h after delivery [45]. This dramatic drop may contribute to sleep disruption and mood changes due to an effect on GABA receptor function. Estrogen levels fall from 6000–30,000 pg/mL to below 40 pg/mL postpartum, thereby impacting serotonin synthesis and receptor sensitivity. Normally, the diurnal cortisol rhythm exhibits a 50–160% increase in the morning hours, which is known as the cortisol awakening response; during postpartum depression, this becomes blunted, with a flatter daytime curve and increased evening levels of >5 μg/dL.

The sudden decline in postpartum hormones, such as oxytocin, significantly influence the neurotransmitter systems involved in regulating mood [46,47]; moreover, a dysregulation in the HPA axis may cause altered responses to stress and metabolic dysfunction [48,49]. Most patients with sleep apnea show elevated levels of prolactin, while low prolactin has been associated with a higher risk for PPD [50,51]. Disturbances in circadian rhythms and melatonin secretion have also been linked to both conditions, highlighting the role of sleep–wake cycle regulation in maternal mental health [52,53,54].

Sleep disruption involves neurotransmitter systems that are crucial for mood regulation, particularly decreasing serotonin, changes in cortisol rhythms, and increased stress reactivity, which have been highly implicated in the pathophysiology of depression [55,56]. Fragmented sleep manifests as irritability, mood instability, and disruptions in mother–infant bonding [57]. Furthermore, sleep fragmentation hurts executive function, working memory, and attention capacity [58]. Structural and functional brain changes due to chronic intermittent hypoxia, identified by neuroimaging, include the areas of the brain associated with mood regulation, such as the prefrontal cortex, amygdala, and hippocampus [59]. Such identified changes include altered neural connectivity, synaptic plasticity, and neurotransmitter systems, which may underpin the increased vulnerability to depression in sleep apnea patients [60,61]. In particular, the dysregulation of neurotrophic factors, including Brain-Derived Neurotrophic Factor (BDNF), is a critical link between intermittent hypoxia and mood disorders. Systemic inflammation and oxidative stress are significant bridging mechanisms between sleep apnea and PPD [62,63].

### 5.3. Inflammatory Pathways

Both disorders demonstrate evidence of increased levels of pro-inflammatory cytokines, including Interleukin-6 (IL-6), Tumor Necrosis Factor-alpha (TNF-α), and C-Reactive Protein (CRP) [64,65]. These inflammatory markers can permeate the blood–brain barrier to impact mood modulation systems [66]. Repeated cycles of hypoxia–reoxygenation during sleep apnea result in the overproduction of Reactive Oxygen Species (ROS), overwhelming antioxidant defense mechanisms [67,68]. This seems to be a self-reinforcing circle, as depression itself can worsen sleep apnea via increasing inflammation and oxidative stress [69,70].

## 6. Diagnostic Challenges

**Figure 3:**
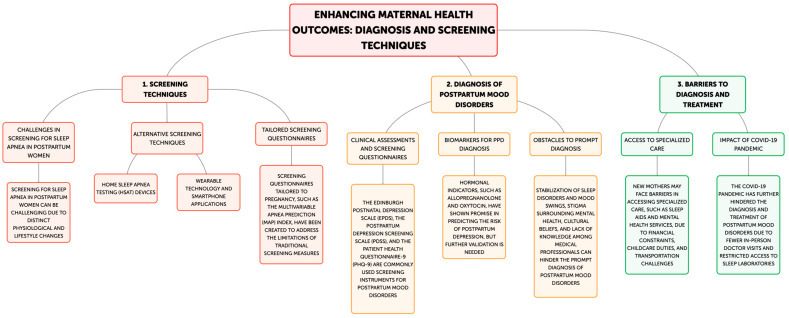
Diagnostic challenges of PPD in women with sleep apnea.

The accurate diagnosis of sleep apnea and PPD among postpartum women requires a deep understanding of the intricacies of various screening techniques, evaluation tools, and diagnostic barriers (Figure 3).

Traditional screening tools, such as the Berlin Questionnaire and the Snoring, Tiredness, Observed Apnea, Blood Pressure, Body Mass Index, Age, Neck Circumference, Gender (STOP-BANG) questionnaire, have focused on general population risk factors and may lack the sensitivity to detect postpartum-specific sleep disorders [20]. While polysomnography is considered to be the gold standard in diagnosis, it has several key logistical limitations for new mothers needing to care for and nurse their children. Home sleep apnea testing devices provide convenience to patients by offering sleep evaluations in familiar environments with measurements of oxygen saturation, respiratory events, and sleep positions [71]. However, Home Sleep Apnea Testing (HSAT) may underestimate sleep apnea severity, especially in mild to moderate cases [72]. Modern techniques include wearable technology and smartphone applications that allow for continuous sleep monitoring, but these also require further validation regarding their diagnostic accuracy in the postpartum population [73]. The adapted Mean Airway Pressure (MAP) index for pregnancy and postpartum conditions seems promising, but larger demographic validation is still needed. PPD screening is generally based on questionnaires that have undergone validation. The most widely used is the Edinburgh Postnatal Depression Scale. This self-reporting tool is a 10-item instrument that has been translated into many languages and cultures [74]. Other instruments include the Patient Health Questionnaire-9 (PHQ-9), with a good reported diagnostic accuracy [75], and the more comprehensive 35-item Postpartum Depression Screening Scale (PDSS) [76]. Success with these instruments is totally dependent on honest self-reporting without bias from cultural perceptions regarding symptom expression. There is also considerable controversy over the optimal timing for screening and how often assessments need to be performed throughout the first postpartum year [77,78]. Other new diagnostic tools under investigation involve the analysis of biomarkers, especially hormonal markers such as allopregnanolone and oxytocin [51]. Although these methods are very promising, they require further validation to become widely used clinically. In addition, the cultural pressures and societal expectations of new motherhood keep many women from talking about their symptoms and seeking help [79]. Limited awareness of this comorbidity among healthcare providers leads to missed opportunities for thorough assessment in both PPD and sleep apnea [80]. Specialized care is another major barrier; very often, financial concerns, childcare responsibilities, and transportation issues get in the way of the needed diagnostic testing for postpartum women [81,82]. The COVID-19 pandemic added to the complexity of such diagnoses by limiting in-person visits and access to a sleep laboratory. While telemedicine has become a proxy for testing in many instances, it faces challenges in replicating the complexities of mood disorder diagnoses and OSA diagnoses.

## 7. Clinical Impact and Developmental Consequences of Sleep Apnea–PPD Comorbidity

The comorbidity of PPD and sleep apnea has critical implications for both maternal and child health outcomes, all the way from immediate postpartum health up to long-term development. Mothers with sleep apnea may feel extremely sleepy and fatigued throughout the day, impacting their sensitive and responsive caregiving abilities. Considering the influences of PPD on emotional and cognitive states, these symptoms result in a lesser reactivity of mothers to infant cues and positive interactions. It has been documented that depressed mothers display a less positive mood during interactions and can struggle to interpret their infants’ emotional signals [83]. Similarly, various research studies conducted in this regard have shown that the infants of PPD mothers, while engaged in face-to-face interactions, exhibit less vocalizing, less positive emotion, and greater avoidance during interactions [84]. Together, maternal sleep apnea and PPD appear to be important factors in reduced infant development across domains. Developmental delays may be related to a lack of intellectual or cognitive stimulation, as well as to parenting interaction and responsiveness.

Longitudinal studies following pairs of mothers and infants during the first two years of life have shown that the children of mothers with postpartum depression (PPD) score lower in assessments of language and cognitive development relative to the children of mothers that do not suffer from PPD [6]. This delay in development seems to correlate specifically with the timing and severity of maternal depression during the postpartum period, where more severe and chronic PPD correlates more strongly with child development [8,16].

Sleep apnea further accentuates these factors by limiting the mother’s potential for either stimulating or rewarding interactions. More vulnerable to disturbance is emotional development, with infants showing increased behavioral problems, lowered emotional regulation abilities, and heightened negative effects. This development may be further exacerbated by several mechanisms. Mothers with both conditions often report problems with regular feeding patterns and breastfeeding, resultant from fatigue and emotional distress [85]. Stress hormones connected with both disorders may be transferred to the fetus and infant, and influence their physiological development, which may be related to later-life health outcomes [86]. Maternal sleep disorders and mood disturbances may also contribute to disturbed infant sleep–wake cycles, promoting later-life sleep disorders associated with developmental morbidity [87]. Consequences also extend beyond postpartum acute health. Cardiovascular health is particularly vulnerable, since both conditions independently contribute to the risk of stroke, coronary artery disease, and hypertension [88]. Chronic intermittent hypoxia in sleep apnea, combined with inflammation from depression, may hasten atherosclerosis and vascular dysfunction. Metabolic health is another concern; sleep apnea is associated with long-term weight retention, while PPD is associated with an increased risk for type 2 diabetes [89]. Long-term cognitive impairment results from the interaction of intermittent hypoxia from sleep apnea with chronic sleep fragmentation, manifesting itself as attention, executive function, and memory deficits [90]. This kind of cognitive impairment might persist and deteriorate along with symptoms of depression. The course of mental health may include an increased risk of recurrent depressive episodes, with sleep apnea likely contributing to the emergence of chronic mood disorders [91]. In the light of the changes in the HPA axis and/or other alterations within the endocrine system, both disorders affect neuroendocrine function [92]. This, in turn, may significantly influence immune function, vulnerability to stress, and reproductive functions. Additional concerns could be related to the potential risk of recurrence in the next pregnancy, with spiraling distal consequences for health [93].

## 8. Therapeutic Strategies and Management Challenges

### 8.1. Medical Treatment

CPAP is still considered to be the gold standard of treatment for sleep apnea; its application in postpartum women, however, presents challenges. These include nocturnal arousals to attend to the infant and discomfort in wearing masks during this sensitive period [94,95]. Adaptation protocols that have been used are auto-titrating devices and gradual initiation protocols, although large-scale validation studies are still required. Other approaches, such as positional therapy and mandibular advancement devices, are less invasive [96,97], although studies on their efficacy are still warranted in this population, which may allow for better adherence. Weight management programs, the other key treatment modality in the approach to sleep apnea, need to be carefully managed in postpartum women. A range of programs need to balance the nutritional requirements for breastfeeding against gradual weight loss objectives [98,99], and, therefore, reflect a highly specialized approach specific to the postpartum period. Psychotherapy and pharmacological interventions have emerged as two evidence-based treatments for PPD. Cognitive–behavioral and interpersonal therapies exhibit a strong efficacy [100], further enhanced by a range of emerging internet-based CBT platforms that increase accessibility [101]. Questions remain, however, about the long-term effectiveness of digital therapy compared to more traditional in-person treatment. SSRIs represent an important component in the management of moderate to severe PPD. A new injectable, brexanolone, showed a promising rapid onset [102]; its accessibility, however, is limited by its high costs and inpatient administration requirements. Safety concerns about the use of antidepressants during lactation also persist, despite drugs like paroxetine and sertraline having determined safety profiles [103]. Non-pharmacological treatments like exercise and bright light therapy are also yielding positive results [24,104] with negligible side effect profiles. However, their comparative efficacy with standard treatments, particularly in more severe illness, still needs to be established [105]. Indeed, a growing number of studies now support the concurrent treatment of both disorders [75]. It has also been observed that the treatment of sleep apnea improves the effectiveness of PPD therapy, while improved mood states tend to promote better compliance with the treatment for sleep apnea.

### 8.2. New Technology for Tailored Management

The technology-assisted monitoring of mood and sleep allows for the continuous collection of data and its analysis, thereby permitting treatment responses in a timelier manner. This monitoring is coupled with mindfulness-based interventions aimed at developing symptom awareness and symptom modulation skills in women. There is promise in the use of modified CBT-I adapted specifically for postpartum women, insofar as it can concurrently target both sleep and mood [106,107,108]. Notable benefits have also been illustrated through partner involvement in treatment, although the actual implementation of this has brought forth a host of challenges, such as those regarding the availability of support and varied forms of family structure [109]. The success of interventions that involve partners has often rested upon various factors, including partner availability, relationship dynamics, and cultural contexts. Thus, stepped-care approaches have emerged as resource-economical interventions that can be gradually escalated in accordance with symptom severity and response [110,111,112,113].

## 9. Current Research Trial and Future Directions

Several relevant clinical trials have addressed the interplay in both directions between sleep apnea and PPD. The SOMNOM study [Sleep Optimization for Mothers with Newborns and Obstructive Apnea Management] evaluated tailored approaches to CPAP management in the postpartum setting [114], while the DREAM trial addressed models of care that incorporate sleep evaluation into comprehensive treatment regimens for PPD [115]. In addition, the University of Pittsburgh’s study investigated bright light therapy with sleep hygiene education, among others [116], and the SNOOZE trial evaluated CBT-I smartphone apps for postpartum women [117]. Advanced technologies in home sleep testing present some promising options for postpartum female screening for sleep apnea [118]. Artificial intelligence in sleep study analysis would go a long way in reinforcing diagnostic accuracy [119,120]. Cognitive CPAP devices with telemonitoring functions may improve treatment monitoring and modification [121]. Novel symptomatic treatments include virtual reality therapy [122], while continuous symptom monitoring is enabled by wearable mood monitoring devices [123]. Non-invasive brain stimulation techniques, to which transcranial magnetic stimulation belongs, are also promising at an early stage, but require larger-scale validation [124,125,126,127,128]. The study of genetic, inflammatory, and hormonal biomarkers could be a basis for finding reliable biomarkers for the two disorders. This is a direction that will surely result in the elaboration of personalized medicine methods, considering lifestyle, microbiome, and genetic polymorphisms. However, it faces problems with its execution and affordability. Different challenges in technology integration should be researched from all aspects [129,130,131,132]. Cost-effectiveness analysis continues to play a critical role in the feasibility of implementation and health access equity ensures that treatment reaches all populations, while data protection for privacy has become highly relevant with the expansion of digital solutions.

## 10. Conclusions

In the field of maternal health care, the combination of PPD and sleep apnea poses a difficult problem. The reciprocal relationship between these disorders has been clarified by recent research, which indicates that different physiological and psychological mechanisms may be at play when they aggravate one another. Important concerns concerning modern screening procedures and therapeutic philosophies are brought up by this interaction. Even while it seems that more postpartum women than previously believed have sleep apnea, diagnosing this condition is still difficult. Identification is complicated by the overlap of symptoms with typical postpartum weariness, and routine sleep investigations may not be feasible for new mothers. Our diagnostic techniques need to be reevaluated considering this circumstance. Could wearable technology or at-home sleep testing provide a workable solution? How can we strike a compromise between postpartum women’s practical limitations and the requirement for a precise diagnosis? Although treatment modalities are changing, there are still concerns regarding their applicability and efficacy in the postpartum setting. Although helpful for treating sleep apnea, CPAP therapy can be difficult for new mothers to adhere to. Resource limitations may prevent CBT-I from being widely used, despite its significant promise in treating depression symptoms and sleep difficulties. How can treatment models be made more accessible and integrated? These co-occurring diseases may have an impact on family dynamics and infant development, in addition to the health of the mother. The extent of postpartum care is a topic of ethical concern when viewed from this wider angle. Should our approach to treatment be more family-centered and holistic? In the future, additional research will be necessary in a few important areas. How can we create screening instruments that are more targeted and sensitive for this population? What part might newly developed technology play in medical diagnosis and care? What effects do socioeconomic and cultural variables have on how these diseases are managed and presented? It is obvious that treating sleep apnea and PPD in postpartum women calls for a multidisciplinary approach as we wrestle with both issues. Collaboration between researchers, politicians, and healthcare professionals can help us to develop more thorough and efficient care plans for new mothers.

## Figures and Tables

**Table 1:** Systematic table.

Authors and Year	Study Type	Sample Size	Population	Groups	Tools/Interventions	Primary Outcomes	Secondary Outcomes	Main Results	Statistical Analysis	Study Limitations	Level of Evidence
Bourjeily et al. (2010) [19]	Cross-sectional study	1,577,632 pregnant women	Pregnant women in immediate postpartum period at a large women’s hospital	Single group divided into snorers and non-snorers	Validated questionnaire for sleep-disordered breathing symptoms and pregnancy outcomes	Association between snoring and pregnancy-induced hypertension, gestational diabetes	Associations with planned cesarean delivery	Frequent snoring: 35% of women	Chi-square tests	Self-reported data	Level III
							Unplanned cesarean delivery	Association with gestational hypertension (OR 2.3, CI 1.4–4.0)	Student’s t-tests	Cross-sectional design	
							Birth weight	Association with preeclampsia (OR 2.22, CI 1.94–2.54)	Multivariate logistic regression	Recall bias	
								Association with gestational diabetes (OR 1.51, CI 1.34–1.72)	Adjusted odds ratios with 95% CI	No objective sleep measurements	
										Single-center study	
Facco et al. (2010) [20]	Prospective observational study	189 pregnant women	Pregnant women receiving prenatal care at Northwestern Memorial Hospital	Single group, assessments performed in early pregnancy (6–20 weeks) and mid-pregnancy (28–37 weeks)	Modified Berlin Questionnaire and Epworth Sleepiness Scale (ESS)	Prevalence of sleep disturbances during pregnancy	Changes in sleep patterns and disorders from early to mid-pregnancy	Mean sleep duration was significantly shorter *p* < 0.001	Chi-square tests for categorical variables	Potential selection bias	Level II
					Women’s Health Initiative Insomnia Rating Scale			Greater frequent snoring *p* = 0.03	Paired t-tests for continuous variables	Reliance on self-reported symptoms	
					Pittsburgh Sleep Quality Index			Restless leg syndrome increased *p* < 0.001	McNemar test for paired proportions	Limited generalizability	
									Multivariate logistic regression	Cross-sectional nature of data collection	
										No objective sleep measurements	
										Single-center study	
Putnick et al. (2020) [21]	Longitudinal cohort study	4.866 pregnant women	Population-based birth cohort of women enrolled in the Upstate KIDS Study in New York State (2008–2010)	Single cohort followed at 4, 12, 24, and 36 months postpartum	Edinburgh Postnatal Depression Scale (EPDS) administered at each time point	Trajectories of maternal postpartum depressive symptoms across the first 3 years postpartum	Associations between trajectories and maternal/child characteristics	Identified 5 distinct trajectories of depressive symptoms:	Chi-square tests	Potential selection bias	Level II
								Low–stable (74.7%)	ANOVA	Limited generalizability	
								Low–increasing (8.2%)	Multiple logistic regression	Possible attrition bias	
								Medium–decreasing (12.6%)	Adjusted for demographic and pregnancy characteristics |—Self-reported symptoms	No clinical diagnoses	
								High–persistent (4.6%)		Limited data on treatment	
										Latent growth mixture modeling	
Redhead et al. (2020) [22]	Cross-sectional study	189 pregnant women	Pregnant women recruited from antenatal clinics in Western Australia between 2012 and 2014	Single cohort assessed in third trimester of pregnancy ≥26 weeks	Berlin Questionnaire for OSA screening	Association between OSA symptoms and depressive symptoms during pregnancy	Impact of sleep quality and other sleep parameters on depressive symptoms	Women reporting OSA symptoms were 8.36 times more likely to have depressive symptoms	Logistic regression	Self-reported symptoms	Level III
					Pittsburgh Sleep Quality Index (PSQI)|			26% of participants had elevated depressive symptoms	Chi-square tests	Single time point assessment	
					Edinburgh Postnatal Depression Scale (EPDS)			Poor sleep quality was associated with increased depressive symptoms	T-tests	No objective sleep measures	
					Hospital Anxiety and Depression Scale (HADS)			Significant association between snoring and depressive symptoms	Multivariate analysis	Possible selection bias	
									Adjusted for potential confounders	Limited generalizability	
										No postpartum follow-up	
Pien et al. (2014) [23]	Prospective cohort study	105 pregnant women	Women recruited from hospital-based practices before 20 weeks gestation in Philadelphia area	Single cohort followed through pregnancy with assessments in each trimester	Polysomnography	Berlin Questionnaire	Relationship with SDB, preeclampsia, and adverse maternal/fetal outcomes	Chronic hypertension, and gestational diabetes were associated with increased risk	Chi-square tests	Single-center study	Level II
						Multiple Sleep Latency Test		Risk factors for OSA during pregnancy	Logistic regression	Relatively small sample size	
						Epworth Sleepiness Scale		Pre-pregnancy BMI and neck circumference were significant predictors	Mixed effects modeling	Possible selection bias	
								Changes in OSA across pregnancy trimesters	Repeated measures ANOVA	Limited generalizability	
								OSA frequency increased from 10.5% in first trimester to 26.7% in third trimester	Adjusted for confounders	Loss to follow-up	
										No postpartum follow-up	
Dennis et al. (2009) [24]	Multisite randomized controlled trial	701 women	Women in the first 2 weeks postpartum identified as high-risk for PPD	Intervention group (receiving telephone-based peer support) vs control group (standard postpartum care)	Edinburgh Postnatal Depression Scale (EPDS)	Prevention of postpartum depression among high-risk women	Maternal satisfaction	Lower risk of PPD in intervention group at 12 weeks postpartum (odds ratio 0.46, 95% CI 0.24–0.62)	Chi-square tests	Self-selected sample	Level I
					Peer volunteer support via telephone		Social support	40% reduction in PPD risk with peer support	t-tests	Unable to blind participants	
					Structured diagnostic interview for depression		Use of health services	Higher maternal satisfaction with peer support	Logistic regression	Possible contamination between groups	
					UCLA loneliness scale		Breastfeeding duration	80% of women would recommend peer support to friends	Intention-to-treat analysis	Reliance on self-report measures	
									Adjusted for baseline variables	Limited generalizability to low-risk women	

## Data Availability

No new data were created for this study.
